# On the hodological criterion for homology

**DOI:** 10.3389/fnins.2015.00223

**Published:** 2015-06-23

**Authors:** Macarena Faunes, João Francisco Botelho, Patricio Ahumada Galleguillos, Jorge Mpodozis

**Affiliations:** ^1^Department of Anatomy, Faculty of Medical and Health Sciences, University of AucklandAuckland, New Zealand; ^2^Grupo Fritz Müller-Desterro de Estudos em Filosofia e História da Biologia, Departamento de Filosofia, Universidade Federal de Santa CatarinaFlorianópolis, Brasil; ^3^Programa de Anatomía y Biología del Desarrollo, Facultad de Medicina, Instituto de Ciencias Biomédicas, Universidad de ChileSantiago, Chile; ^4^Laboratorio de Neurobiología y Biología del Conocer, Departamento de Biología, Facultad de Ciencias, Universidad de ChileSantiago, Chile

**Keywords:** amniote pallium, amygdala, cortex, dorsal ventricular ridge, epigenesis, evolution, organization

## Abstract

Owen's pre-evolutionary definition of a homolog as “the same organ in different animals under every variety of form and function” and its redefinition after Darwin as “the same trait in different lineages due to common ancestry” entail the same heuristic problem: how to establish “sameness.”Although different criteria for homology often conflict, there is currently a generalized acceptance of gene expression as the best criterion. This gene-centered view of homology results from a reductionist and preformationist concept of living beings. Here, we adopt an alternative organismic-epigenetic viewpoint, and conceive living beings as systems whose identity is given by the dynamic interactions between their components at their multiple levels of composition. We posit that there cannot be an absolute homology criterion, and instead, homology should be inferred from comparisons at the levels and developmental stages where the delimitation of the compared trait lies. In this line, we argue that neural connectivity, i.e., the hodological criterion, should prevail in the determination of homologies between brain supra-cellular structures, such as the vertebrate pallium.

## Introduction: The problem of homology

The concept of homology has long been implicitly used by biologists, as comparison has been the basis of our classification of the natural world at least since Aristotle (Russell, 1916; Nordenskiold, 1928). Nevertheless, the study of structural correspondence moved to the foreground (Russell, 1916; Coleman, 1971) in the first half of the nineteenth century, when biology emerged as an independent science and morphology became its core discipline. By comparing the structure of living beings, early morphologists sought the laws that govern form and function. Similar structures meant similar plans (*Gestalt*) or similar generational rules (*Bildung*), and the comparison of anatomy and embryology were a means to discover them. Therefore, biological similarity was explained by sameness of type, much like similar structures in minerals. In this typological context, Richard Owen defined a “homolog” as “the same organ in different animals under every variety of form and function” (Owen, 1843; Panchen, 1994).

Biology was radically transformed at the second half of the nineteenth century by the theory of evolution (Ruse, 1999; Bowler, 2003). The large amount of data gathered from comparative anatomy and embryology by earlier morphologists was one of the most important sets of evidence presented by Darwin (1859) to support his theory, and it was subsequently re-interpreted in light of the new theoretical framework. The archetype of early morphologists was replaced by the ancestor, and the concept of homology was reappraised in genealogical terms (Haeckel, 1874). As stated by Karl Gegenbaur, a leading morphologist converted to evolutionism, “the theory allowed what previously had been designated as *Bauplan* or *Typus* to appear as the sum of structural elements of animal organization which are propagated by means of inheritance” (cited in Coleman, 1976). The explanation for sameness changed from shared organizational rules to shared genealogy, and the “homolog” became defined as “the same trait in different lineages due to common ancestry” (Lankester, 1870).

Although typological and genealogical concepts of homology entailed different views of *sameness*, from a practical point of view, both concepts involved the same operational criteria to define it (Wagner, 1994; Bolker and Raff, 1996; Griffiths, 2007; Hall, 2007). In both cases, homologies could only be inferred by comparing features of the ontogeny and/or the structure of the trait among organisms. However, comparisons of different features, i.e., the use of different homology criteria, often conflict with each other. The rise of experimental embryology at the end of the nineteenth century, and the following advances in cell biology and classical genetics, nourished the expectation that the discovery of developmental mechanisms shared by different lineages would yield an absolute biological criterion for homology. Yet, the many advances in embryology and genetics failed to achieve this. The lack of a unified criterion has persisted obstinately since the origins of evolutionary biology (Darwin, 1859, p. 532) and cell biology (Wilson, 1894), and was thoroughly exposed by De Beer (1971) in his classic paper entitled “Homology: an unsolved problem”. The main conclusions drawn by de Beer were:

**Table:** 

*(i)*	*“… correspondence between homologous structures cannot be pressed back to similarity of position of the cells of the embryo or the parts of the egg out of which these structures are ultimately differentiated.”*
*(ii)*	*“… homologous structures can owe their origin and stimulus to differentiate to different organizer-induction processes without forfeiting their homology.”*
*(iii)*	*“… characters controlled by identical genes are not necessarily homologous.”*
*(iv)*	*“… homologous structures need not be controlled by identical genes, and homology of phenotypes does not imply similarity of genotypes.”*

The problem of *which homology criteria to choose* is perhaps particularly complicated in the field of neuroscience. The structural complexity of the nervous system and its interactions with sensory and motor organs offer multiple possible criteria, and in more than a few instances different criteria disagree (Campbell and Hodos, 1970). This means that anatomical, embryological, physiological, and behavioral features are not always conserved together. For example, in different animals, neurons can have similar connectivity (or *hodology*), neurochemistry and function but display different morphologies and ion channel densities (e.g., Purves and Lichtman, 1985; Marder and Goaillard, 2006) or develop from different embryonic precursors (e.g., Glover, 2001). In the same way, similar behaviors can be conserved despite changes in the underlying neural circuits (e.g., Newcomb et al., 2012).

Even though de Beer's problem remains unsolved (see for example Weiss and Fullerton, 2000; True and Haag, 2001; Kawasaki et al., 2005; Schierenberg, 2005), there is currently an assumption—implicit or explicit—that homology problems must be addressed by developmental genetics. Many recent events, such as the appearance of DNA sequencing tools, the concept of regulatory genes in eukaryotes and the *in situ* analysis of genetic expression, converged to renew the hopes of finding an absolute criterion for homology. Indeed, comparative developmental genetics has produced some of the most important achievements in evolutionary biology in the last decades, resulting in profound consequences to the concept of homology.

## Saint-Hilaire's lobster and the dorsoventral patterning genes: The reductionist appraisal of an organismic statement

A good example reflecting the historical implications of the developmental genetics approach is the 1990s revival of Geoffroy Saint-Hilaire's hypothesis of the morphological homology between the dorsal side of vertebrates and the ventral side of arthropods. Around 30 years before Owen articulated his definition of homology, the pre-evolutionary anatomist Geoffroy Saint-Hilaire was already seeking a formal criterion for designating homologs (which he called “analogs”). In the preliminary discourse of the first tome of his “Philosophie Anatomique” he offers the following criterion: “The only generality to be applied to the species is given by the position, the relations and dependences between the parts, that is, by what I embrace and designate as connections” (Saint-Hilaire, 1818). By proposing a unity of composition, or “*unité de système dans la composition et l'arrangement des parties organiques*” (“unity of system in the composition and arrangement of organic parts”) for all animals, Saint Hilaire defied the ruling notion of the time, put forward by his colleague Georges Cuvier. According to Cuvier, every animal followed the body plan of one of the four *embranchements* of the animal kingdom: *vertebrata, mollusca, articulata, and radiata* (Cuvier et al., 1817). With his *loi des connections* (law of connections), according to which the connections held between homologous organs in different animals remain constant, Saint-Hilaire established various homologies between vertebrates and invertebrates, which resulted in the indignation of Cuvier. One of his audacious proposals was that the body plan of a lobster, an *articulata*, was the same as that of a *vertebrata*, only with its dorsoventral axis inverted (Saint-Hilaire, 1998 [1822]). This lead to a great controversy that most historians agree was won by Cuvier.

Molecular embryologists reappraised Saint-Hilarie's hypothesis based on the inverted similarity of genes expressed in the dorsal and ventral sides of the embryos of fruit flies and frogs (Arendt and Nubler-Jung, 1994; de Robertis and Sasai, 1996). The finding of a conserved set of molecular interactions led them to postulate the inversion of the dorsoventral axis during early chordates evolution and therefore to recognize the homology between vertebrate and arthropod nervous and digestive systems. The fact that Saint Hilaire's hypothesis—edified on the basis of comparative anatomy—only came to be reconsidered after more than 150 years, following findings in the field of molecular biology illustrates the impact of the developmental/genetic criteria of homology in current biology. The discovery of common DNA sequences and molecular interactions across animal phyla revealed an unexpected new level of conservation. A number of evolutionary developmental biologists took these and other similar findings with caution and postulated the term “deep homology” to refer to the conservation of a “genetic regulatory apparatus” in morphologically disparate traits among distantly related species (Shubin et al., 1997; Hall, 2003). However, many others took those cases as exemplars for a new reductionist agenda: to elucidate the conservation of molecular processes in early ontogeny in order to resolve problematic homologies.

Nevertheless, considering the difficulties faced by developmental criteria when determining homology, we could pose two counterfactual questions: Could we confidently ascertain that the neural system of arthropods and vertebrates are non-homologs if they had different molecular mechanisms of dorso-ventral axis specification? Certainly not, since variations in developmental mechanisms at early ontogenetic stages occur remarkably often. Inversely, could we confidently ascertain as homologs any neural and digestive systems that are specified by the same early developmental mechanism? Neither, since common developmental mechanisms can generate different structures.

## A competing organismic-epigenetic view of homology

Why does developmental genetics face such hindrances when attempting to provide an absolute criterion for homology? We believe this to be the consequence of one of the most prominent characteristics of living beings: they are dynamic systems organized into multiple levels (Jacob, 1970; Mayr, 1982). The hegemony usually granted to developmental and gene expression-centered homology criteria results from ontological assumptions that collapse the levels of organization and the embryological history of organisms into their lower levels and first stages of development. These assumptions are the consequence of a reductionist and preformationist view of living beings according to which development consists of the execution of a genetically-coded building program. Organisms are regarded in this framework as mosaics of ontogenetically independent components whose structural properties are determined not through their mutual interactions during development, but by the accomplishment of their corresponding segment of the genetic program (Carroll, 2005; Hoekstra and Coyne, 2007). If this were the case, then the identity of a trait would be solely given by gene expression patterns during its development.

If we assume that living beings are dynamically changing systems that exist through continuous interactions between their components in the epigenetic course of development, then we cannot reduce the identity of all traits to a particular ontogenetic stage, such as early development, or a particular level of organization, such as the molecular level. The components of a living system can (and constantly do) change without the identity of the system nor the coherence with its environment being lost, and these changes can occur at some levels of its organization without producing changes in other levels, during both ontogeny and phylogeny (Bertalanffy, 1962; Maturana and Varela, 1973; Maturana and Mpodozis, 2000)^1^. It is the continuous historical (moment to moment) realization of their organization—i.e., of the relations held between their organic components at different structural levels—what confers to organisms their identity at any stage of ontogeny.

Three relevant consequences follow the adoption of this organismic/epigenetic approach to living beings:

- Neither developmental nor genetic comparisons can supply an absolute criterion for determining homology. Given the systemic nature of living organisms and the epigenetic nature of their development, the recurrence of traits between generations does not imply the recurrence of genetic nor developmental processes, because a given ontogenetic state can be constituted by different sets of components and attained by different developmental trajectories.
- When establishing a homology, both the level of organization and the ontogenetic stage to be considered must be in agreement with the delimitation of the compared trait. Inasmuch as there is no privileged level or stage in the realization of living organization, delimiting the object is part of the establishment of a homology. The delimitation of a trait is the distinction of a particular organization, a particular set of relations held between components within the organism, and therefore it is defined by the observer and is not intrinsic to the composition of the living system (Wimsatt, 1972; Striedter, 1999; Griesemer, 2000; Winther, 2006). In the same way, the establishment of a homology is defined by the observer because it is the distinction of the same set of relations within two individuals or lineages (Maturana, 2002). The more reliable criteria to assess a homology will be those aspects of the compared trait that are most structurally restricted to change while the organization that defines the trait is conserved.
- The phylogenetic explanation is independent of the establishment of a homology (Amundson and Lauder, 1994). Considering that inheritance is the repetition of a process and not the transmission of a trait (Maturana and Mpodozis, 2000; Oyama et al., 2001), whether a homologous trait is present in the most recent common ancestor of the compared species (what has been called a “true” homology) or is the result of parallel evolution (“latent” homology) is relevant for its explanation, but irrelevant for its definition (Arendt and Reznick, 2008). In both cases the homology results from the recurrence of a historical, epigenetic process^2^.

## A long-standing homology problem in the nervous system: The case of the amniote telencephalon

The pallium is the dorsal part of the vertebrate telenchephalon, and in mammals its most prominent structure is the six-layered isocortex. In diapsids (reptiles and birds), however, most of the pallium is composed of the *dorsal ventricular ridge* (DVR), which is organized into nuclei. Homologies between the pallia of amniotes have been subject of much debate over the last 20 years. The controversy has been previously reviewed by others (e.g., Reiner et al., 2005) and will be presented here only briefly. The first tract-tracing studies that began to reveal the organization of the sensory collothalamic projections (i.e., those sensory projections reaching the thalamus through a relay in the midbrain) to the avian DVR led to the proposal of a possible homology between nuclei in the avian anterior DVR and specific layers in mammalian temporal isocortices (Karten, 1969). Further studies continued to reinforce this notion by showing striking similarities in the overall organization of sensorimotor circuits; from the midbrain and thalamic structures (which become homologized by extension, e.g., Major et al., 2000) to the intra DVR circuits and the targets of their descending projections (e.g., Wild et al., 1993; Wang et al., 2010; Ahumada et al., 2015).

**Figure 1:**
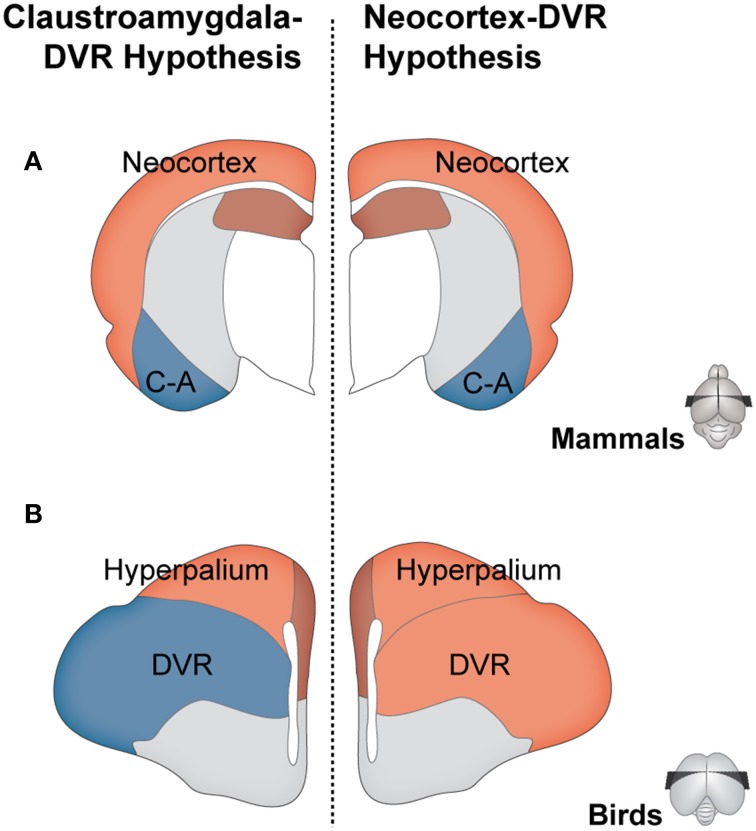
**Competing hypotheses regarding the homologies between mammalian and diapsid pallia**. Schematic representation of coronal sections of the brains of a mammal **(A)** and a bird (**B**, as example of a diapsid). According to the claustroamygdala-DVR hypothesis (left side), the avian DVR and hyperpallium are homologous to the mammalian claustroamygdalar complex (blue), and neocortex (light red), respectively. According to the isocortex-DVR hypothesis (right side), the avian DVR and hyperpallium are both homologous to the mammalian isocortex (light red).

Twenty-five years after it was first enunciated, this “isocortex/DVR hypothesis” was challenged by the proposal of the “claustroamygdala/DVR hypothesis.” First, based mostly on work on the connections of the reptile forebrain, Bruce and Neary (1995) put forward the hypothesis that the mammalian homolog of the DVR was the basolateral amygdala (Figure 1). Even though this hypothesis has received some further support from hodological evidence (e.g., Novejarque et al., 2004; Guirado et al., 2005), what truly fueled the debate was the later work on homeobox gene expression patterns during development (Reiner et al., 2005; Bruce, 2012). Different authors proposed the amygdala and/or claustrum and endopiriform nucleus as mammalian homologs to the DVR (Striedter, 1997; Fernández et al., 1998; Puelles et al., 2000; Aboitiz et al., 2003). Thus, the earlier isocortex vs. claustroamygdala controversy became a debate between hodology and development/gene expression. More recently, this debate has moved to a new phase, primarily due to novel evidence showing that specific components of the avian DVR express layer-specific isocortical markers (Dugas-Ford et al., 2012; Chen et al., 2013; Suzuki and Hirata, 2013) and that there is a common pattern of gene-expression between the DVR and the hyperpallium (the widely-accepted diapsid homolog to the striate cortex, see Figure 1B) during development (Jarvis et al., 2013). These new data are seen as key support to the isocortex hypothesis (Karten, 2013; Reiner, 2013), and thus –much like the case of Saint Hilaire's Lobster– the focus of the debate has shifted to development/gene expression grounds.

We consider the reduction of the problem to a case of development/gene expression similarities to be intrinsically misdirected. Whatever their embryonic or adult patterns of gene expression, the hodological similarities of the diapsid DVR with the mammalian isocortex and basolateral amygdala remain the same. Homologies of gene-expression or cell types do not imply homologies of the supra-cellular structures containing them, and homologies of embryonic domains of gene expression certainly do not imply homologies of the resulting adult structures. Levels and stages of comparison should not be intermingled.

Accordingly, we think that the question about the identity of the adult diapsid DVR can only be focused at the level where the traits “neocortex” and “amygdala” are defined, which is the supra-neuronal level. What defines the identity of a supraneuronal structure is the set of relations it holds with the rest of the nervous system, which in an adult nervous system conform sensorimotor correlations of neural activity. The sensorimotor correlations that define the neocortex and amygdala are attained by the functional interconnectivity between different neuronal groups and sensory and motor organs, and not by properties intrinsic to any of them. The aspects more restricted to change, and thus the most useful as homology criteria, are those directly related to the connectivity that maintain these sensorimotor correlations. These can include neuronal morphology, neurochemistry, and most importantly, hodology. Therefore, we consider that the way to settle the issue of the diapsid DVR is to further unveil the organization of the circuits it is involved in.

## Conclusions

Most contemporaneous philosophers of science accepted the assumption that scientific concepts should be dealt with in their social and historical context (Dupré, 2012)^3^. To recognize that scientific concepts are determined by the operations and practices employed to define them, and not by the intrinsic properties of the object, is an important premise in the present debate about the definition of homology. To search for an absolute or “biological” criterion of homology, able to explain sameness across time and levels, is unfeasible and unnecessary. The sound establishment of a homology means the sound comparison and description of sameness in a scientific domain. Like a sound experiment, it shall survive to different theories or explanations (Griffiths, 2007). In other words, whatever our explanation for homology is, it will not deny the sameness in the connectivity of the vertebrate visual system described by Cajal, the ontogeny of the mandibular arches described by Meckel, or the synteny of genes described by genomics studies.

The acknowledgement of the organismic-epigenetic nature of living beings accounts for the incongruences in the search for an absolute homology criterion and for the necessity to consider the ontogenetic and organizational delimitation of the compared trait when attempting to establish a homology. Homologies are not to be determined according to the fulfillment of a unique criterion, nor the largest amount of criteria, but of the most appropriate criteria according to the level of analysis where the identity of the trait is defined. From this organismic-epigenetic perspective, the identity of the adult nervous system, when considered from a high level of its organization, is defined by the interactions that allow the coordination of the activity of different neuronal populations and motor and sensory systems, i.e., its functional connectivity. Focusing on the hodological criterion rescues a key aspect of living beings: that they are processes. As such, the identities of their components are given by their interactions with the rest of the organism during the process of life, or what Saint-Hilaire called *connections.*

### Conflict of interest statement

The authors declare that the research was conducted in the absence of any commercial or financial relationships that could be construed as a potential conflict of interest.

## Footnotes

^1^The evolution of actin filaments is a good example. The DNA sequences used by the cell to the production of actin proteins in eukaryotes are so different from the sequences used to produce MreB in bacteria that their homology had been ignored until the tertiary structures of the proteins were revealed, showing that lower levels of organization have changed while the structure necessary for the process of dynamical polymerization has been conserved (Erickson, 2001; Colavin et al., 2014).

^2^De Beer (1971) and Hall (2007) have also proposed a congruency between “true” and “latent” homologies, but their analyses differ to and have points of disagreement with ours, as they appeal to the phylogenetic continuity of the “developmental basis” (i.e., developmental genes) for parallel evolution to produce “true” homologous structures.

^3^The epistemology of the concepts of *gene* and *species* illustrates well the power of this new kind of philosophical analysis in biological sciences. *S*pecies and *genes* mean different things for different sets of scientists and several philosophers of biology tend to assume that they are defined and transformed by the practice (Dupré, 2012; Godfrey-Smith, 2013; Griffiths and Stotz, 2013).
